# Supplementary material for: National Trends in Suicides and Male Twin Live Births in the US, 2003 to 2019: An Updated Test of Collective Optimism and Selection in Utero
Source: Twin Res Hum Genet. Author manuscript; Available in PMC 2025 Jun 15. (PMC11178679; doi:10.1017/thg.2023.49)
Supplement: figures [file NIHMS1992810-supplement-figures.docx]

Supplement Figure 1: Plot of percent monthly change in suicides among females aged 15-49 years in the US, from January 2003 to December 2019.

Supplement Figure 2: Plot of autocorrelation function (ACF) up to 12 month lags of monthly male twin ratio after specifying autoregressive parameters at lags 1 and 12 (AR 1, 12).

Supplement Figure 3: Residual series (after removal of autocorrelation) of percent monthly change in suicides among women aged 15-49 years, from January 2003 to December 2019, in the US. Initial 12 observations consumed in autocorrelation parameter modelling.

Supplement Table 1: Time-Series results for monthly male twin ratios from January 2003 to December 2019, as a function of exposure to de-trended residuals of percent monthly change in suicides among females aged 15-49 years and autocorrelation parameters.

| Variable | Coefficient | Standard Error |
| --- | --- | --- |
| Constant | 0.034 | 0.000*** |
| Autoregressive parameter: AR 1 | 0.076 | 0.077 |
| Autoregressive parameter: AR 12 | 0.688 | 0.052*** |
| Differencing | None | |
| Exposure lag (de-trended residuals of percent monthly change in suicides among females aged 15-49 years) |  |  |
| 2 | 0.0000 | 0.0005 |
| 3 | 0.0000 | 0.0006 |
| 4 | 0.0002 | 0.0007 |
| 5 | -0.0001 | 0.0006 |
| 6 | -0.0012 | 0.0005* |

*p < 0.05; two-sided test.

**p < 0.01; two-sided test.

***p < 0.001; two-sided test.
